# Effectiveness of a School-Based ‘Tobacco Free’ Intervention on Adolescents’ Knowledge and Exposure to Second Hand Tobacco Smoke - A Multiphase Study

**DOI:** 10.31557/APJCP.2019.20.12.3533

**Published:** 2019

**Authors:** Ashwini Rao, Unnikrishnan B, Prasanna Mithra, Nandini M, Ramya Shenoy, Nikita Rungta

**Affiliations:** 1 *Department of Public Health Dentistry, Manipal College of Dental Sciences, *; 2 *Department of Community Medicine, Kasturba Medical College, Mangalore, *; 3 *Department of Biochemistry, Kasturba Medical College, Mangalore, A Constituent Unit of Manipal Academy of Higher Education (MAHE), Manipal, India. *

**Keywords:** Adolescent, cotinine, environmental exposure, randomised controlled trial, second hand tobacco smoke

## Abstract

**Background:**

There is no safe level of exposure to second hand tobacco smoke (SHS). The World Health Organization has stressed that 100% smoke-free environments are the only effective way to protect the population from the harmful effects of exposure to SHS.

**Design:**

A multiphase study with a descriptive cross-sectional questionnaire phase 1 and a phase 2 cluster randomized controlled trial (RCT), conceptualized to determine the effectiveness of a school-based ‘tobacco free’ health education intervention on adolescents’ knowledge and attitude towards SHS.

**Methods:**

Baseline assessment will include a questionnaire followed by estimation of salivary cotinine levels. The experimental arm will receive the ‘tobacco free’ intervention, which includes 40 min health education session delivered once a week for 3 consecutive weeks. Participants will also be given ‘take home brochures’ every week containing messages on the effects of tobacco and how to make their homes smoke-free. The sample of 250 participants, for the Phase 2 RCT, with salivary cotinine levels of > 0.1 ng/mL will be selected from the participants of the Phase 1 study. The effect of the intervention will be quantitatively assessed by estimating the salivary cotinine levels after the intervention. Participants in the control arm will receive conventional standard health education once.

**Conclusion:**

This research will help in assessing if there is any change in the salivary cotinine levels and the knowledge, attitude and behaviour scores after the health educational intervention and help in developing an effective school-based ‘tobacco free’ intervention program which could be incorporated into the school curriculum. This study has received the Public Health Research Initiative (PHRI) Research Grant of Rs. 18,99,205 and is registered with the Clinical Trial Registry of India (CTRI) with number CTRI/2018/09/015706 (Registered on 13/09/2018). Ethical approval has been obtained from The Institutional Ethics Committee (No.17021 dated 13 march 2017).

## Introduction

“*Tobacco is the single most preventable cause of death in the world today. During the 21st century, it is estimated to kill one billion*”(WHO, 2008). Cigarettes are found to contain about 1.5% nicotine by weight, which produces 1–2 mg of bioavailable nicotine per cigarette. Therefore, inhaling tobacco smoke is the main source of nicotine exposure for the general population (CDC, 2018). 

Cotinine, a major metabolite of Nicotine is a useful biomarker with a half life of about 16 hours and can be used to distinguish tobacco users from non- users (Raja et al., 2016).

Although many countries of the World have passed laws making workplaces, public places, and restaurants smoke-free, still millions of children and adults continue to breathe second hand tobacco smoke (SHS) (CDC, 2017). According to the World Health Organization (2018), the health of almost half of the world’s children is affected by exposure to environmental tobacco smoke (ETS).

SHS exposure has been linked to a variety of serious diseases in adults as well as in children, such as coronary heart disease, lung cancer, breast cancer, respiratory symptoms and illnesses, otitis media and during pregnancy causes pre-term low birth weight deliveries (WHO., 2018).

There is no safe level of exposure to second hand smoke. Even brief exposures can be harmful. Moreover, studies (Farkas et al., 2000; Öberg et al ., 2011) have even shown that “*children exposed to SHS are more likely to become smokers themselves when they grow older compared to those unexposed*”.

This paper describes the study protocol for a multi-phase study, which has been conceptualized to develop and evaluate a ‘*tobacco free*’ intervention program to reduce children’s exposure to Second Hand Tobacco Smoke. 

*Rationale*

Although educating children might be an effective method of reducing tobacco prevalence not only among the present adults but also among the adolescents who are our future adults (Alwan et al., 2010; Siddiqi et al., 2010; Zafar Ullah et al., 2013; Eakin et al., 2014; Loke and Mak, 2015), literature search revealed gaps in research pertaining to tobacco free intervention among adolescents, resulting in the conception of this study. 

**Figure 1:**
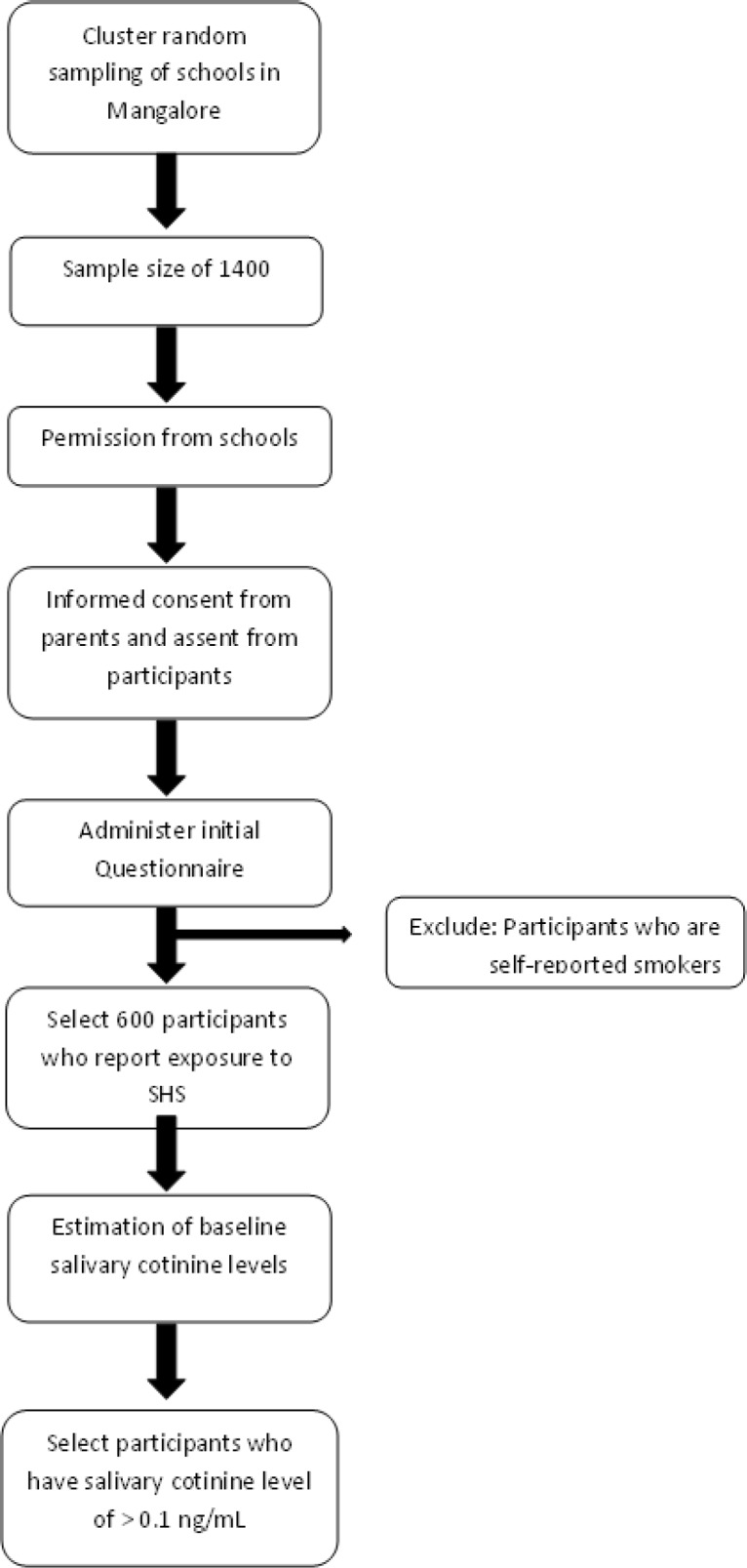
Flow of Participants in Phase 1

**Figure 2:**
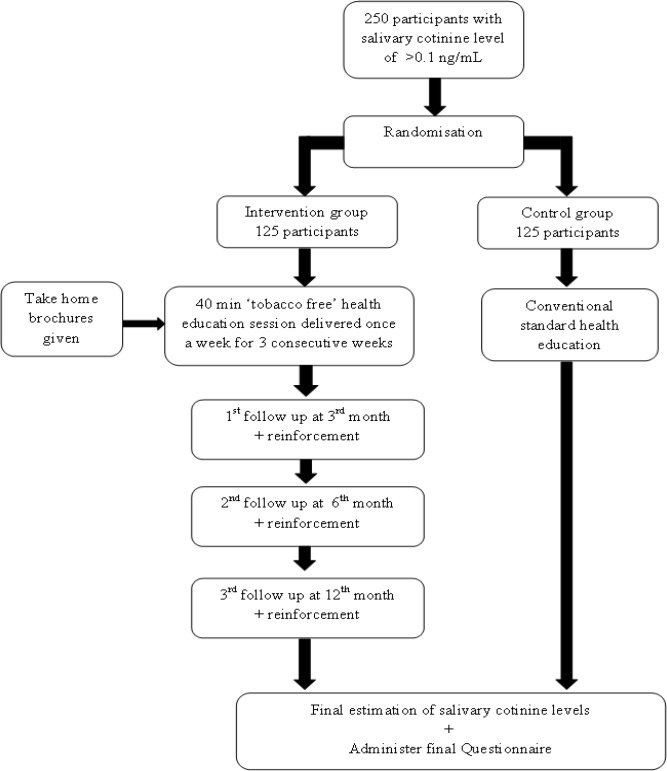
Flow of Participants in Phase 2

## Materials and Methods

*Study setting*

The study will be conducted among 12 year old school children of Mangalore, India. 

*Broad objective:*

To contribute toward national efforts to protect children from harmful effects of SHS, the objective of this study is to develop effective school-based intervention to reduce exposure and improve adolescents’ knowledge and attitude towards Second Hand tobacco Smoke and to promote smoke free homes.

*Specific objectives*

To assess exposure to second hand tobacco smoke among 12 year old school children.

To carry out a school based ‘*tobacco free*’ educational intervention program among 12 year old school children.

To assess the Salivary Cotinine level as well as the knowledge, attitude and avoidance behaviour towards second hand tobacco smoke among 12 year old school children, before and after carrying out an intervention. 

*Study design and registration*

This will be a multiphase study using multiphase sampling technique among adolescents in Mangalore, India. The first phase will be a descriptive cross-sectional study. A cluster random sampling of all the schools in Mangalore will be done to obtain the required sample size of 1400. A questionnaire will then be administered to the 12 year olds in the selected schools, who fulfil the inclusion criteria. Data will be analysed and those students who report exposure to second hand tobacco smoke will be selected and their salivary cotinine level checked until we get a sample of 250 subjects with salivary cotinine levels of > 0.1 ng/mL (Avila-Tang et al., 2012). These will form the sample for the second phase of the study which will be a randomized controlled trial. (Figures 1 and 2). 

The protocol follows SPIRIT guidelines (Chan et al., 2013) and includes all items from the World Health Organization Trial Registration Data Set (WHO., 2019) [Appendix 1]. The trial has been registered with the Clinical Trial Registry of India (CTRI) with number CTRI/2018/09/015706 (Registered on 13/09/2018).

*Sample size calculation*

*For phase 1: (Cross-sectional study)*

The sample size estimation was done using the formula:

$n=\frac{{\left(Z_{1-a/2}\right)}^{2}\mathrm{PQ}}{d^{2}}$

where: 

Z_1-α/2_ = 1.96 (when α is 5% at 95% confidence limits)

P= Proportion of the event in the population (29%) (Singh et al., 2011)

Q=1-P (0.71)

d= Acceptable margin of error (3%)

With a design effect at 1.5 and a non-response at 5%, the final sample size was calculated to be 1400 for the Phase 1 study.

*For phase 2: (Randomized controlled trial)*

The sample size calculation for Phase 2 randomized controlled trial was done using G Power 3.1.2. We calculated a total sample size of 250 with125 in the control group and 125 in the intervention group, 90% power, an Effect size of 0.3, confidence interval at 95% and attrition at 10%.

*Recruitment and eligibility*

The study is now recruiting and the criteria for eligibility are as follows, 

*Inclusion criteria for phase 1 study*

1. All Schools in Mangalore who have primary and secondary schools in the same campus.

2. Those schools who consent to participate in the study

3. Students whose parents give written informed consent and they themselves give informed assent.

*Exclusion criteria for phase 1 study*

1. Participants who are self-reported smokers.

*Inclusion criteria for phase 2 study*

1. Inclusion criteria: Participants with salivary cotinine levels of > 0.1 ng/mL

2. Exclusion criteria: Participants whose sibling is already a participant in the study

*Study procedure*

*Phase 1*

The first phase will be a descriptive cross-sectional study. A cluster random sampling of all the schools in Mangalore will be done to obtain the required sample size of 1,400. A questionnaire will then be administered to the 12 year olds in the selected schools, who fulfil the inclusion criteria. 

*Questionnaire*

Demographic data will be recorded. Socioeconomic status will be assessed using the BG Prasad Scale (Prasad., 1961) modified considering the base of Consumer Price Index (CPI) for 2017 (Singh et al., 2017). The questionnaire will have two components, one to assess children’s exposure to SHS using Questionnaire 1 which has 5 questions (Lin et al., 2010). The second component, Questionnaire 2, is a 25 item questionnaire (Gharaibeh et al., 2011) to determine Children’s knowledge, attitude and behaviour towards second hand smoke. Knowledge will be assessed using 10 items, Attitude with 5 items, Avoidance behaviour towards second hand smoke will be assessed using 5 items and Self-efficacy of avoidance will be assessed using 5 items. This assessment will be carried out at the start of Phase 1 and at all 3 follow-up time points in Phase 2.

The questionnaire will be administered to 10 individuals and reliability measured using the Cronbach’s coefficient alpha. A high coefficient indicates that the items are consistently measuring the same underlying construct.

Data will be analysed and those students who report exposure to second hand tobacco smoke will be selected and their salivary cotinine level checked.

*Salivary Cotinine level estimation*

Children’s exposure to SHS will be determined by measuring their salivary cotinine levels twice, one at the end of Phase 1(baseline) and second at the 3rd follow-up, i.e., at 12 months after the intervention. Salivary cotinine is a sensitive biochemical marker which is strongly associated with exposure to SHS. It has a half-life of 16 hours and is a widely recognized marker for detecting both active and passive smoking. 

Saliva collection: Saliva will be collected not before at least 60 minutes after a major meal. Participant will be asked to rinse mouth thoroughly with water 10 minutes before sample is collected. The head is tilted forward, allowing the saliva to pool on the floor of the mouth. Whole saliva will be collected in a labelled vial by unstimulated passive drool. After collection, sample will be refrigerated within 30 minutes, and frozen at or below -20ºC within 4 hours of collection. 

Salimetrics^®^ High Sensitivity Salivary Cotinine Quantitative Enzyme Immunoassay Kit will be used for cotinine estimation in saliva (Salimetrics., 2018). There will not be any personal identifiers on the vials sent to the laboratory and individual results will not be disclosed.

The participants who have salivary cotinine levels of > 0.1 ng/mL will be selected and they will form the sampling frame for the second phase of the study which will be a randomized controlled trial.

*Phase 2*

This phase will be a randomized controlled trial, with a sample size of 250, 125 participants in the intervention group and 125 in the control group.

Random allocation: Sequence generation will be done using the table of random numbers. We will adopt the process of first identifying potential individual participants in each cluster, recruiting these participants and then do cluster randomisation. Allocation concealment will be done using sequentially numbered, opaque, sealed envelopes (SNOSE). The study will be outcome assessor blinded.

*Intervention*

The intervention consists of 40 min ‘tobacco free’ health education session delivered once a week for 3 consecutive weeks by the principal investigator (PI) (AR). The first session will address tobacco and its adverse effects. The second session will be on laws regarding tobacco in India and the World and the third session will be on how to prevent exposure to tobacco. Teaching methods include lectures, demonstrations, discussions, storytelling and quiz. Children are also given take home brochures every week that contains messages on the effects of tobacco and how to make their homes smoke-free.

There will be 3 follow up time points. The first follow up will be at the 3rd month, second follow up at the 6th month and the third follow up at 12 months after the intervention. On the first day of each follow up, the PI will reinforce the key points of the intervention and also discuss with the students regarding their experiences at home and the challenges they faced. The timeline is given in Appendix 2. Participants in the control arm will receive conventional standard health education once, whereas those who report tobacco use will be encouraged to participate in tobacco counselling.

*Study outcomes*

To assess if there is any change in the salivary cotinine levels and the knowledge, attitude and behaviour scores of the participants after a ‘tobacco free’ health educational intervention.

To develop effective school-based intervention to reduce exposure and improve adolescents’ knowledge and attitude towards Second Hand tobacco Smoke and to promote smoke free homes.

*Data analysis plan*

Analysis will be done based on both the Intention to Treat and Per Protocol analysis. The data will be entered into the SPSS software (IBM Corp. Released 2011. IBM SPSS Statistics for Windows, Version 20.0. Armonk, NY: IBM Corp.) and analysed. Descriptive statistics will be presented and sub group analysis will be done wherever needed. The level of significance will be kept at 0.05 with 95% confidence levels. For the phase 1 study, Chi square test will be done for categorical variables and Independent sample t test will be done to compare means. Logistic regression will be done to obtain the adjusted odds ratio. For the phase 2 study, Paired sample t - test will be done.

*Ethical aspects*

Ethical approval has been obtained from The Institutional Ethics Committee (No.17021 dated 13 march 2017). Written informed consent from parents and informed assent from the participants will be obtained by the principal investigator prior to recruitment. Confidentiality of the participants will be maintained at all times. The saliva collected will be completely utilized for laboratory evaluation and no biological specimen will be stored for further use.

In conclusions, although children might be exposed to SHS in a lot of places, the primary source of SHS exposure is in their homes (Zafar Ullah., 2013; WHO., 2015). As the prevalence of adult smokers increases, so does the number of children who breathe air polluted by tobacco smoke, particularly at home. This will be a first of its kind study to be done in this part of the World. The World Health Organization has stressed on the need for “*implementing complete smoke-free environments as the only effective way to protect the population from the harmful effects of exposure to SHS*” (WHO., 2015). Educating children about the ill effects of second hand smoke will empower them to decide and take action so as to choose to breathe in an atmosphere unadulterated with toxic chemicals released from tobacco smoke. The child might also take home and share this message thus helping in reducing tobacco use not only among the present adults but also among the adolescents who are our future adults. India is a signatory to the “*WHO Framework Convention on Tobacco Control (FCTC), which calls for measures to protect people from SHS*” (WHO., 2019). The outcome of this research will help in developing effective school-based intervention to improve adolescents’ knowledge and attitude towards Second Hand Smoke and to promote smoke free homes.

*Funding*

This study has received the Public Health Research Initiative (PHRI) Research Grant of Rs. 18,99,205 as an extramural fund set up by Public Health foundation of India (PHFI) in collaboration with Science and Engineering Research Board (SERB, A Statutory Body under Department of Science and Technology (DST), Government of India). The funding agency has not influenced the design of this study and will not influence its execution, analyses, interpretation of the data. 

*Authors’ contributions*

AR conceived the study. AR, UB, PM, NM and RS participated in the study design and NR helped with modules and permissions. AR is the grant holder and UB and RS have provided statistical expertise in the trial design. All authors contributed to refinement of the study protocol and approved the final manuscript.
